# Large organellar changes occur during mild heat shock in yeast

**DOI:** 10.1242/jcs.258325

**Published:** 2021-08-11

**Authors:** Katharina S. Keuenhof, Lisa Larsson Berglund, Sandra Malmgren Hill, Kara L. Schneider, Per O. Widlund, Thomas Nyström, Johanna L. Höög

**Affiliations:** 1 Department for Chemistry and Molecular Biology, University of Gothenburg, Gothenburg 41390, Sweden; 2 Department of Microbiology and Immunology, The Sahlgrenska Academy at the University of Gothenburg, Gothenburg 41390, Sweden; 3 Department of Psychiatry and Neurochemistry, Institute of Neuroscience and Physiology, The Sahlgrenska Academy at the University of Gothenburg, Gothenburg 41390, Sweden; 4 Department of Medical Genetics, Cambridge Institute for Medical Research, Cambridge CB2 0XY, UK

**Keywords:** Electron microscopy, Ultrastructure, Budding yeast, Heat shock, Organelles

## Abstract

When the temperature is increased, the heat-shock response is activated to protect the cellular environment. The transcriptomics and proteomics of this process are intensively studied, while information about how the cell responds structurally to heat stress is mostly lacking. Here, *Saccharomyces cerevisiae* were subjected to a mild continuous heat shock (38°C) and intermittently cryo-immobilised for electron microscopy. Through measuring changes in all distinguishable organelle numbers, sizes and morphologies in over 2100 electron micrographs, a major restructuring of the internal architecture of the cell during the progressive heat shock was revealed. The cell grew larger but most organelles within it expanded even more, shrinking the volume of the cytoplasm. Organelles responded to heat shock at different times, both in terms of size and number, and adaptations of the morphology of some organelles (such as the vacuole) were observed. Multivesicular bodies grew by almost 70%, indicating a previously unknown involvement in the heat-shock response. A previously undescribed electron-translucent structure accumulated close to the plasma membrane. This all-encompassing approach provides a detailed chronological progression of organelle adaptation throughout the cellular heat-stress response.

## Abstract (precis)

**Summary:** Exposure to mild heat shock leads to large quantifiable changes in the cellular ultrastructure of yeast, showing the involvement of multivesicular bodies in the heat-shock response and the apparition of novel structures.

## INTRODUCTION

Increasing the temperature activates the heat-shock response in a cell. This is an ancient and evolutionarily conserved transcriptional program that results in reduced expression of genes involved in protein biosynthesis pathways and increased expression of genes encoding heat-shock proteins (Verghese et al., 2012). The heat-shock response is also activated by other types of stressors, e.g. oxidative stress, exposure to heavy metals, fever and protein conformational disorders (Morimoto and Westerheide, 2010).

Heat shock in budding yeast (*Saccharomyces cerevisiae*) is extensively used as a model to study neurodegenerative human diseases (Winderickx et al., 2008; Kaliszewska et al., 2015), where inclusion bodies of aggregated misfolded proteins accumulate at specific sites in the cytoplasm and nucleus (Takalo et al., 2013; Chung et al., 2018). Examples of such diseases are Alzheimer's, Huntington's and Parkinson's (Tenreiro et al., 2013). The processes involved in the genesis of these diseases are heavily influenced by the temporal and spatial interactions of a protein, both of which can be suitably studied using budding yeast (Debburman et al., 1997; Schirmer and Lindquist, 1997).

In eukaryotes, the heat-shock transcription factor (HSF, a family of DNA-binding proteins that regulate gene expression at the transcription level) is responsible for the induction of heat-shock genes. It exists in its inactive form during non-stress conditions and is activated when there is an accumulation of destabilised, misfolded proteins in the cell. HSF binds DNA and initiates transcription (Morimoto, 1993). The heat-shock-induced changes in transcription and translation ensure that the cell is capable of maintaining proteostasis and metabolism when experiencing temperature stress (Mühlhofer et al., 2019). Although many of these molecular mechanisms of the cellular heat-shock response have been widely studied, the wide-reaching structural and architectural effects of such a temperature change are often neglected because attention is focused on the system of interest, e.g. the transcriptome, proteome and/or the behaviour of misfolded proteins (Shi et al., 1998; Verghese et al., 2012).

In this study, we have investigated the structural adaptations that cells undergo when subjected to a mild 38°C heat shock. A heat shock that is comparable with that used when studying protein quality control as well as temperature-sensitive mutants. Using electron microscopy of high-pressure frozen cells, we obtained ultrastructural information on nearly every cellular organelle and substructure, complementing in detail the important insights gained by fluorescent microscopy in studies of several candidate proteins (Meaden et al., 1999; Lewandowska et al., 2006; Spokoini et al., 2012; Escusa-Toret et al., 2013; Meyers et al., 2016; Gao et al., 2017). To gain a nanometre-resolution map of cellular alterations prompted by heat shock that is as comprehensive as possible, a minimum of 100 cells were imaged for each time point throughout a continuous 90 min exposure to 38°C. The time course and subsequent imaging were performed in triplicates, yielding a total of 2143 images analysed for an overview that comes as close to a screen as possible using electron microscopy. The resulting temporal map of cellular restructuring reveals that heat shock induces major morphological changes to the internal architecture and composition of the cell, and that more organelles than anticipated are involved in this process.

## RESULTS

### Heat shock has wide-reaching effects on cell structure

**Fig. 1.**
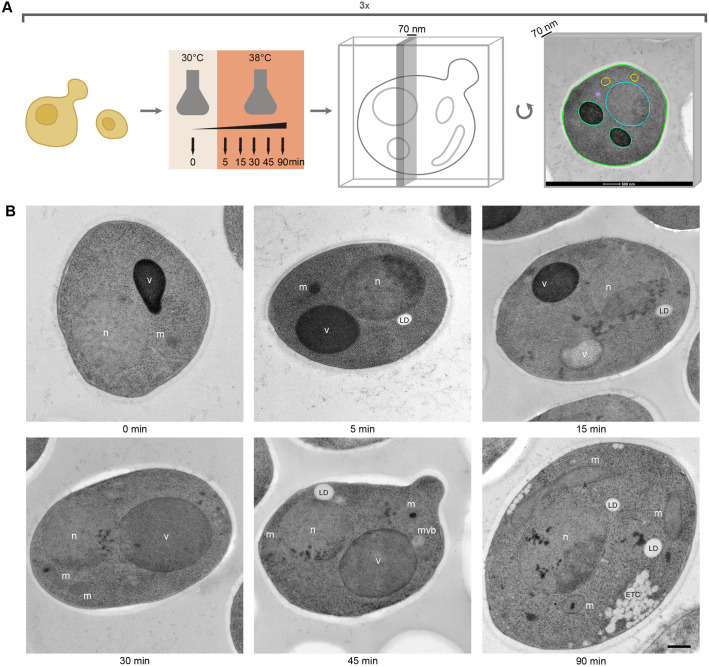
**Revealing ultrastructural changes during a mild heat shock using a transmission electron microscopy screening method**. (A) Cartoon of the experimental procedure: cells were grown at 30°C and heat shocked at 38°C for 0, 5, 15, 30, 45 and 90 min. Cells were then high-pressure frozen, freeze-substituted and sectioned at 70 nm for electron microscopy. Each heat-shock experiment was carried out in triplicate and at least 100 images were collected of each sample, resulting in a total of 2143 images analysed. Size and morphology of all clearly visible organelles were modeled (coloured circles in rightmost image) and quantified to reveal known and unknown effects of exposing yeast cells to heat shock. (B) Example images show that large morphological changes and cellular reorganisation occurred during heat shock. Furthermore, a new phenotype that we call electron translucent clusters (ETCs) appear during heat shock. n, nucleus; v, vacuole; m, mitochondrion (label next to organelle); mvb, multivesicular bodies (label next to organelle); ld, lipid droplet. Scale bar: 500 nm.

We used electron microscopy to study the ultrastructural changes occurring in yeast during a mild heat shock over a time course of 90 min (Fig. 1A). Samples of yeast cultures were collected at 30°C and after a 5, 15, 30, 45 or 90 min shift to 38°C. These samples were cryo-immobilised using high-pressure freezing followed by freeze substitution for best possible morphological preservation (Moor, 1987; Perktold et al., 2007; Höög et al., 2016). Samples were then sectioned into 70 nm thin sections. At least 100 random images were collected at a magnification where the entire cell could be visualised in detail (9300×, pixel size 1.1 nm). The whole experiment was repeated three times.

Observations of cells subjected to such temperature stress revealed unexpectedly large changes in cellular architecture (Fig. 1B). Organelles, such as lipid droplets (LDs), seemed to increase both in number and size (Fig. 1B, see 5 and 45 min). Electron-dense content, which has also been shown to be protein aggregates in the case of the nucleus (Panagaki et al., 2021), was observed in both the nucleus and mitochondria (Fig. 1B, see 15, 30, 45 and 90 min). Previously undescribed electron-translucent clusters (ETC) appeared often in close proximity to the plasma membrane (Fig. 1B, see 90 min). However, the most prominent difference was the altered morphology of the vacuole. In heat-treated cells, the vacuole appeared to change both in texture, electron density and size (Fig. 1B, compare 0 and 30 min; Fig. S1). To further investigate these cellular adaptations, we progressed to outline all distinguishable organelles and to quantify their number and area.

### Vacuoles and the cell as a whole increase in size throughout heat shock

**Fig. 2.**
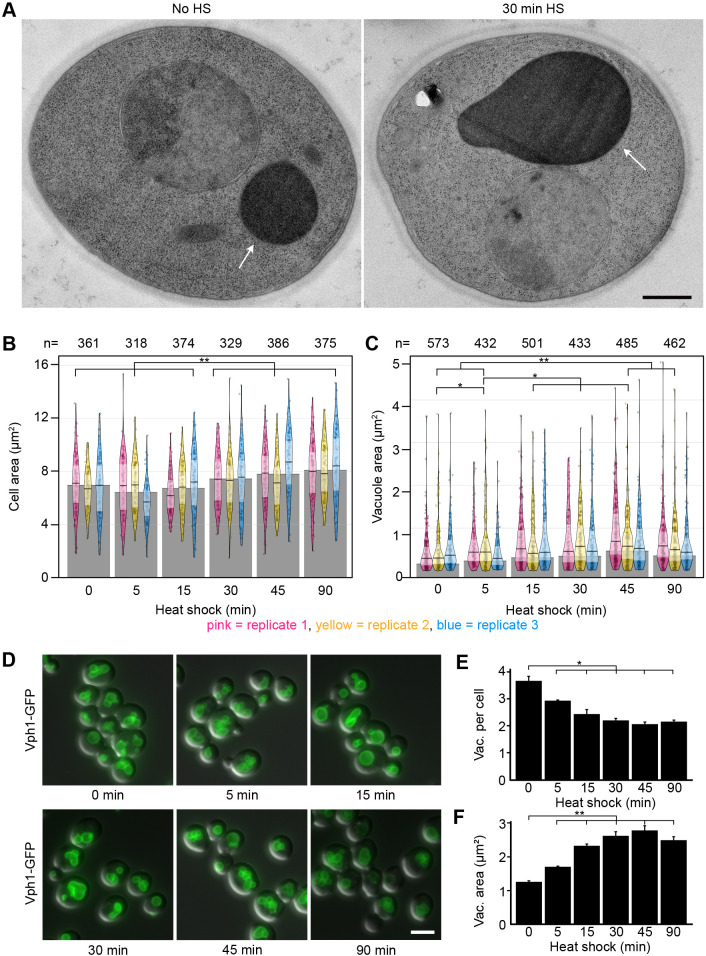
**Cells and vacuoles increase in size.** (A) Cells with vacuoles (arrow) before and after 30 min of heat shock. Scale bar: 500 nm. (B) Cell area in electron micrographs of thin sections. On average, the cell size increases by 19% over the course of 90 min. The pink, yellow and blue shapes represent the three separate replicates. The width represents the number of measurements within a certain range of values, one point represents one measurement. Solid horizontal line and inference bands are median±i.q.r. Grey bars represent the median of all data points within one timepoint. (C) Vacuoles in electron micrographs of thin sections. On average, the vacuole size increases by 48% over the course of 90 min. (D) Cells expressing Vph1-GFP heat shocked at indicated time points. Scale bar: 5 µm. (E) Quantification of vacuole number per cell from cells in D, *n*>200 per replicate and time point. Data are mean±s.e.m. (F) Quantification of vacuole size from cells in D, *n*>200 per replicate and time point. Data are mean±s.e.m. All timepoints were statistically analysed individually. *P<0.05, **P≤0.01.

Outlining 2143 plasma membranes over the six different time points revealed that the average cell area increased by 19% over the 90 min heat shock (Fig. 2A,B). Notable growth occurred between 15 and 30 min, as the first three timepoints all differ significantly from the last three.

The vacuole often occupied the most amount of space in the cell, but during heat shock it grew even more compared with the cell volume (*n*=2866 vacuoles; Fig. 2C). Vacuoles increased in size, peaking after 45 min of heat shock at an area 69% larger than their original size (Fig. 2C). After this, they shrunk slightly but by 90 min still remained 48% larger than before heat shock.

As electron microscopy sections are too thin to contain a whole vacuole, and may not accurately represent the number of large organelles in the cell, we also investigated their change in terms of numbers and shape throughout stress using fluorescence microscopy. Cells expressing Vph1-GFP (Fig. 2D), a subunit of the vacuolar ATPase V_0_ present in the vacuolar membrane (Hecht et al., 2014) were used for this purpose. Although the number of vacuoles decreased by 41% over the course of 90 min (Fig. 2E), at 45 min of heat shock they were 2.2 times as large as those in the control. After 90 min the vacuole was still twice as large as before heat shock (non-heat shocked 1.26±0.05 μm^2^, *n*=614; 90 min heat shock 2.49±0.18 μm^2^, *n*=624; Fig. 2F). This shows that vacuoles probably respond to heat shock by fusing and creating few large organelles rather than many smaller vacuoles, while increasing in net vacuolar volume, which can be obtained by multiplying the average number of vacuoles in a cell by the average size of a vacuole at a specific timepoint. Furthermore, a change to their internal morphology was also observed.

### Vacuolar texture and electron density change in response to heat shock

**Fig. 3.**
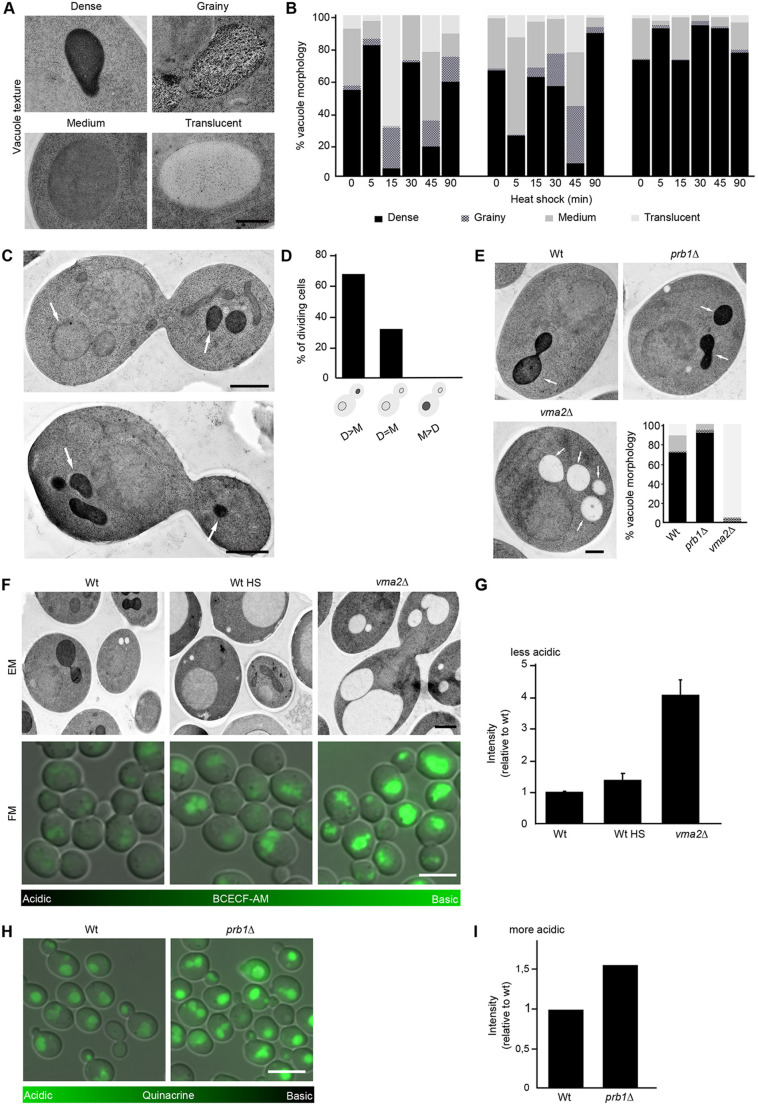
**Heat shock affects vacuolar morphology and acidity.** (A) Gallery of different vacuolar electron densities. Scale bar: 500 nm. (B) Quantification of vacuolar electron density during heat shock. More than 100 vacuoles per time point were analysed from three biological replicates. (C) Electron microscopy (EM) images of dividing wild-type cells grown at 30°C. Arrows point to vacuoles. Scale bars: 1 µm. (D) Quantification of electron density of vacuoles in mothers (Ms) and their daughters (Ds) during cell division, *n*=53 dividing cells. (E) EM images of wild type, *prb1*Δ and *vma2*Δ grown at 30°C, and quantification of vacuolar electron density, *n*=181 (wild type), 150 (*prb1*Δ) and 274 (*vma2*Δ) vacuoles from a minimum of 100 cells. Greyscale for bottom right panel as in B. Arrows point to vacuoles. Scale bar: 500 nm. (F) EM and fluorescence microscopy (FM) of indicated strains. Heat shock (HS) is 45 min at 38°C. The fluorescent pH-sensitive probe BCECF accumulates in yeast vacuoles and shows vacuolar pH. The more fluorescence, the more basic the vacuolar pH. Scale bars: 1 µm for EM; 5 µm for FM. (G) Quantification of vacuolar BCECF fluorescence mean intensity. More than 30 vacuoles were analysed from three biological replicates. Graph shows mean±s.d. fluorescence intensity relative to wild type. (H) Fluorescence microscopy of indicated strains. The fluorescent pH-sensitive probe quinacrine accumulates in yeast vacuoles and shows vacuolar pH. The more fluorescence, the more acidic the vacuolar pH. Scale bar: 5 µm. (I) Quantification of vacuolar quinacrine fluorescence mean intensity. 100 vacuoles were analysed per strain.

A striking variation in electron density of the vacuolar lumen was observed with electron microscopy. In logarithmically growing non-stressed yeast cells, vacuoles are often very electron dense. After heat shock, the vacuoles were often electron translucent and sometimes contained a grainy texture. Vacuoles were therefore classified into four major categories according to their appearance: dense, grainy, medium or translucent (Fig. 3A). The electron density of vacuoles fluctuated strongly throughout the time course in each experiment, showing lower electron densities when exposed to stress and a recovery to darkly stained vacuoles at 90 min (Fig. 3B).

When comparing vacuoles of dividing cells, daughter cells had either a higher or equal vacuolar electron density than their mother (Fig. 3C,D, *n*=53 dividing cells). A previous study showed that upon cell division in yeast, the daughter cell has a more acidic vacuole than its older mother cell (Hughes and Gottschling, 2012). We thus hypothesised that lower electron density in vacuoles could be due to a deacidification of that organelle. The effect of vacuolar pH on the electron density of the samples was investigated using cells from a *vma2*Δ strain (Fig. 3E). Vma2 is a subunit of the V1 domain of the vacuolar H^+^ ATPase and its deletion causes an increased vacuolar pH compared with its usual pH of 5-6.5 (Li and Kane, 2009), this high pH also reduces vacuolar protease activity (Nakamura et al., 1997). As opposed to wild-type cells, almost all vacuoles in *vma2*Δ cells displayed the electron-translucent phenotype, demonstrating that increased pH leads to altered vacuolar staining (Fig. 3E, *n*=181 wild-type and 274 *vma2*Δ cells). To determine whether vacuoles deacidify during heat shock, we used the pH-sensitive vacuolar probe BCECF-AM, which displays increased fluorescence at increased pH (Plant et al., 1999; Hughes and Gottschling, 2012). As a positive deacidification control, BCECF-AM staining also confirmed the expected elevated pH in the *vma2*Δ mutant (Fig. 3F,G). Vacuoles exposed to 45 min heat shock fluoresced brightly, compared with those of cells grown at 30°C, confirming that heat shock causes elevated internal pH in vacuoles (Fig. 3F,G). Furthermore, cells of the deletion mutant *prb1*Δ, where the gene *PRB1* encoding a vacuolar protease is deleted, showed more electron-dense vacuoles than wild type (Fig. 3E, *n*=150). In fluorescence microscopy images of cells stained with quinacrine, an indicator for acidity, vacuoles had higher fluorescence than wild-type cells (Fig. 3H,I). Accordingly, *prb1*Δ cells stained using BCECF-AM had lower fluorescence than wild type (Fig. S1B). Therefore, using our electron microscopy sample preparation protocol, changes in vacuolar pH can potentially be observed as altered electron density of the vacuolar lumen. To summarise, vacuoles are affected by heat shock in several ways: a decrease in number, an increase in size and increased luminal pH.

### Nucleus and mitochondria change in size and develop electron dense content throughout heat shock progression

**Fig. 4.**
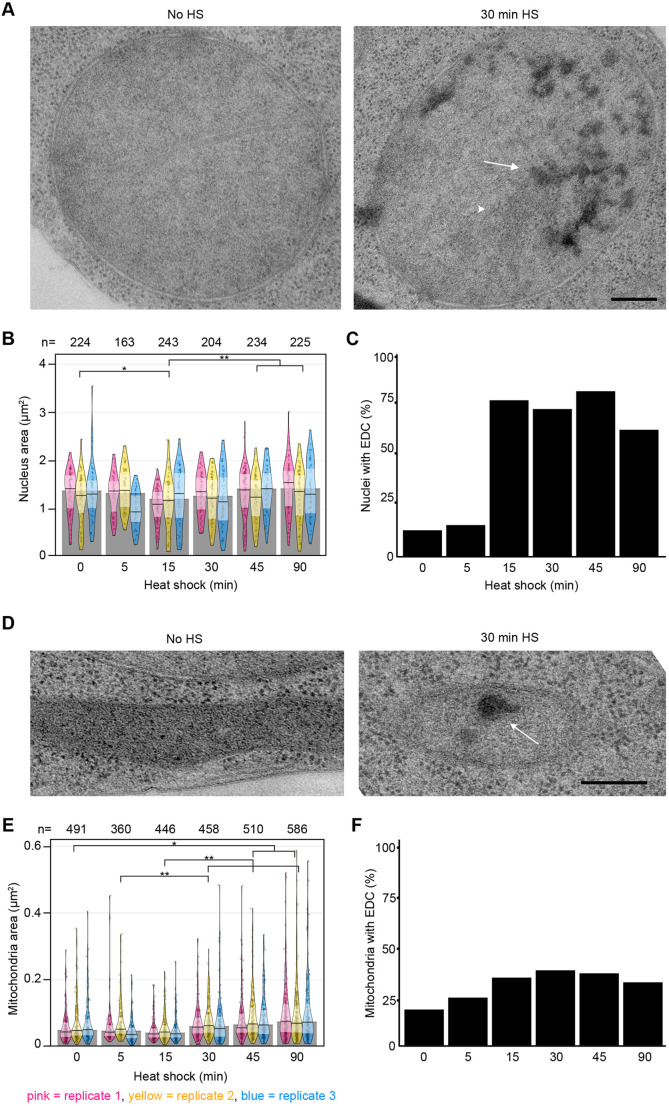
**Nucleus and mitochondria change in size and morphology.** (A) Nucleus morphology before and after 30 min heat shock, as electron-dense content (EDC, arrow) appears next to the nucleolus (arrowhead). Scale bar: 250 nm. (B) Nucleus area in electron micrographs of thin sections. The pink, yellow and blue shapes represent the three separate biological replicates. The width represents the number of measurements within a certain range of values; one point represents one measurement. Solid horizontal line and inference bands are median±i.q.r. Grey bars represent the median of all data points within one timepoint. (C) Proportion of nuclei with EDC throughout heat shock. *n* values are the same as those in B. (D) Mitochondria morphology before and after 30 min heat shock, as an EDC (arrow) appears. Scale bar: 250 nm. (E) Mitochondria area in electron micrographs of thin sections. On average, the mitochondria increase in size by 52% over the course of 90 min. (F) Proportion of mitochondria with EDCs throughout heat shock. *n* values are the same as those in E. All timepoints were statistically analysed individually. *P<0.05, **P≤0.01.

The nucleus was also affected by the increase in temperature (Fig. 4A). Surprisingly, a significant shrinkage (11% smaller than before heat shock) occurred 15 min after temperature shift (Fig. 4B). The nucleus then recovered to the same approximate size as before. Nuclear morphology was also altered and electron-dense content (EDC, arrow in Fig. 4A) became more prominent as heat shock progressed. The proportion of nuclei containing EDC increased rapidly with the onset of heat shock, coinciding with the decrease in size, but had decreased again slightly by 90 min (Fig. 4C). EDC is distinct from nuclear structures such as the nucleolus and chromatin (Hyde, 1965; Karreman et al., 2009; Thelen et al., 2021); at 90 min of heat shock, 90% of EDC in the nucleus was outside the nucleolus (Fig. S2).

As one cell section can contain several mitochondria (Fig. 4D), 2851 mitochondria were analysed (Fig. 4E). Similar to the nucleus, mitochondria were only 75% of their size in untreated cells after 15 min of heat shock and were significantly different from all time points thereafter. However, eventually a total increase in mitochondrial area of 52% after 90 min was noted. The number of mitochondria stayed relatively constant (Fig. S3). The proportion of mitochondria enclosing EDC followed a similar trend to the nucleus, albeit with a less dramatic increase (Fig. 4F). Thus, during heat shock, both nuclei and mitochondria vary in size throughout and also accumulate structures visible as EDC by electron microscopy.

### MVBs and LDs increase in size during heat shock

**Fig. 5.**
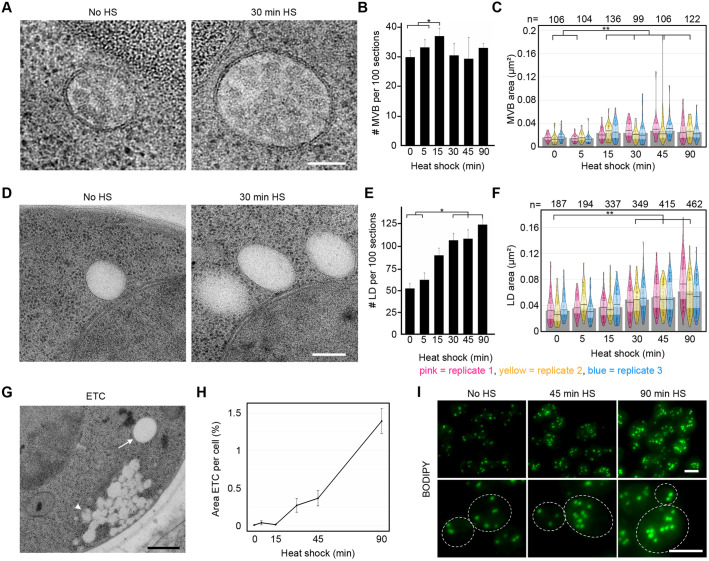
**MVBs and LDs increase in size and a new phenotype appears.** (A) MVB morphology before and after 30 min of heat shock. Scale bar: 100 nm. (B) Number of MVBs (mean±s.e.m.). (C) Area of MVBs increased by 73%, on average. The pink, yellow and blue shapes represent the three separate biological replicates. The width represents the number of measurements within a certain range of values; one point represents one measurement. Solid horizontal line and inference bands are median±i.q.r. Grey bars represent the median of all data points within one timepoint. (D) LD morphology before and after 30 min of heat shock. Scale bar: 200 nm. (E) Number of LDs, increased by factor 2.4, averaged over three sets with error bars corresponding to s.e.m. (F) Area of LDs increased by 85%, on average. Graph description as in C. (G) Electron translucent clusters (ETCs) (arrowhead) next to an LD (arrow). Scale bar: 500 nm. (H) Area of the cell section that was covered with electron-translucent clusters over time in heat shock, *n*=2143 cells. (I) Maximum projections of fluorescent images of cells stained with BODIPY for visualisation of LDs. Scale bars: 2 µm. All timepoints were statistically analysed individually. *P<0.05, **P≤0.01.

Two of the smallest cellular organelles, MVBs (Fig. 5A) and LDs, displayed the greatest alteration in size over the heat shock time course, with the sizes of both organelles increasing dramatically. The number of MVBs fluctuated during the time course, and after 15 min the cells contained 24% more MVBs before heat shock (Fig. 5B). At the 15 min timepoint, MVBs also significantly differed in size from the control (Fig. 5C); after 90 min they had increased in size by 73% (Fig. 5C).

LDs (Fig. 5D) also increased dramatically in number, and peaked in cells subjected to 90 min of heat shock, where they were found 2.4 times as frequently as in the control group (Fig. 5E). The first significant difference in size was observed after 30 min; by 90 min they had increased in size by 85% compared with their equivalent in untreated cells (Fig. 5F). In brief, temperature stress leads to a general increase in the size and number of these small organelles.

### Electron-translucent clusters appear quickly after heat shock initiation and increase throughout

Previously undescribed grape-like clusters of non-membrane enclosed electron-translucent material appeared in heat shock (arrowhead; Fig. 5G). We named them electron-translucent clusters (ETCs) and could clearly distinguish them from electron-translucent LDs (arrow; Fig. 5G), which are larger, clearly delimited from the cytoplasm and never appear in such large clusters. In addition, LDs were most often observed close to the nucleus (Fig. 5D, Fig. S4), whereas ETCs were often found in close proximity to the plasma membrane (Fig. 5G, Fig. S4). The proportion of the cell area occupied by ETCs steadily increased during the heat shock time course (Fig. 5H). After 90 min at 38°C, an average of 1.4% of the cross-sectional of the cell area was occupied by ETCs, compared with barely having been present before heat shock (when 0.006% of the area was occupied by ETCs, *n*=361 cells).

To investigate whether the content of ETCs and LDs was similar, we stained neutral lipids prominent in LDs (Murphy, 2001) using BODIPY, and compared fluorescence in untreated and heat-shocked cells (Fig. 5I). The fluorescence increased in a manner that corresponds well with the larger and more common LDs, but was not located near the plasma membrane where the ETCs were found in electron micrographs, indicating that the composition of ETCs differs from that of LDs. Therefore, throughout the heat-shock time course, ETCs of an unknown substance increasingly aggregate within the cell near the plasma membrane.

### Physical interaction between vacuole and nucleus increases during heat shock

Not only are organelles of the cell influenced by heat shock as individual components, but their interactions with each other are also affected. Organelles can communicate by forming areas of direct physical contact, called membrane contact sites (MCSs), enabling the exchange of ions, lipids and signals between the organelles (Gottschling and Nyström, 2017). Although some MCSs have been more intensively studied (Pan et al., 2000; Kornmann et al., 2009; Hönscher et al., 2014), MCSs have been identified between almost every cellular organelle (e.g. Liu et al., 2017). The high-resolution information provided by electron microscopy was used to quantify MCSs between nucleus and vacuole and between vacuole and mitochondria throughout the heat-shock time course. An MCS was defined as membranes from two organelles in proximity to each other and not separated by any cytosolic ribosomes. Both the number and lengths of contact sites were quantified. When multiplying the frequency of contact sites with their average absolute length, the potential for interaction between the two organelles can be estimated. To differentiate whether the increased length of contact sites is due to the enlarged organelles or a specific response to heat shock, we calculated the contact site fraction (CSF). This measurement was calculated by expressing the MCS length in relation to the circumference of the adjoining organelles. The CSF was then used to calculate the expected length of MCS as the organelle size varied. When there is a difference between the expected and measured lengths of the MCS, the response is heat-shock specific.

**Fig. 6.**
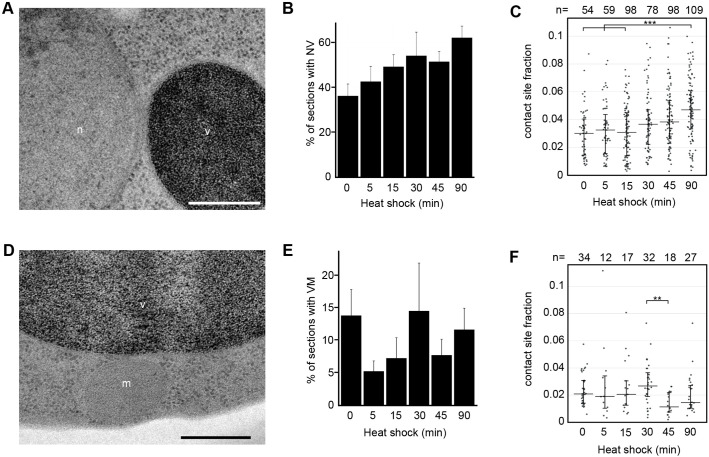
**Membrane contact sites are influenced by heat shock.** (A) Contact site between nucleus (n) and vacuole (v). Scale bar: 50 nm. (B) Percentage of sections containing both nucleus and at least one vacuole with a contact site between the two (NV). Data are mean±s.e.m. of three biological replicates, *n*=1033. (C) Length of contact site in relation to circumference of nucleus and vacuole (median±i.q.r.), *n*=496 MCS, pooled from three biological replicates. (D) Contact site between vacuole (v) and mitochondrion (m). Scale bar: 50 nm. (E) Percentage of sections containing minimum one vacuole and minimum one mitochondrion with a contact site between the two (VM). Data are mean±s.e.m. of three biological replicates, *n*=1347 sections. (F) Length of contact site in relation to circumference of vacuole and mitochondria (median±i.q.r.), *n*=140 MCS, pooled from three biological replicates. All timepoints were statistically analysed individually. **P≤0.01, ***P≤0.001.

Contact sites between the nucleus and vacuoles (Fig. 6A) were not significantly more frequent (Fig. 6B). However, the CSF had increased by a total of 57% after 90 min of heat shock (*n*=1033 sections, Fig. 6C), differing significantly from the beginning of the time course. The absolute length of the nucleo-vacuolar contact sites also became longer throughout heat shock (Fig. S5A), and exceeded the expected value for the increase in size. The interaction potential of MCSs between nucleus and vacuoles more than doubles between the 0 min and 90 min time points, increasing the likelihood of a contact site being established by twofold. This suggests a specific response to heat shock.

The absolute number of contact sites between vacuole and mitochondria (Fig. 6D) fluctuated strongly, both between time points and sets (Fig. 6E). Despite a significant decrease in CSFs between 30 and 45 min, when vacuoles are at their largest, the size of contact sites between vacuole and mitochondria did not significantly change (Fig. 6F, Fig. S5B) overall. The measured lengths of MCSs were equal or close to the expected value for their organelle size. The interaction potential between the two organelles changes in the same way as does the CSF (Fig. S5C,D). In conclusion, we find that a 90-min heat shock appears to specifically increase inter-organellar contact between vacuoles and the nucleus, but the size of the physical interaction between mitochondria and vacuoles largely follows their change in size.

### A map of large architectural changes to the cell during heat shock reveals dynamic changes in organellar size

**Fig. 7.**
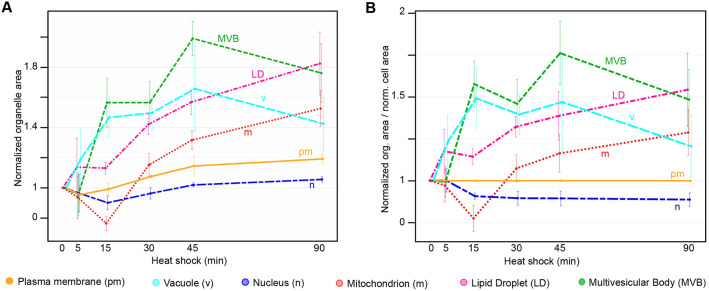
**Relative and comparative changes in organelle sizes.** (A) Change in individual organelle area over heat shock with starting size normalised to 1. Data are mean±s.e.m. of the three biological replicates. (B) Change in organelle area in relation to cell area at the respective time point with starting sizes normalised to 1. Data are mean±s.e.m. of the three biological replicates calculated by dividing average organelle size by average cell size at the respective timepoint.

One could get the impression that the cell and its organelles respond to heat stress with a general size increase (Fig. 7, Fig. S6A,B). This could be due to the effort made by the cell to prevent macromolecular crowding, which influences diffusion within the cell as well as protein folding and aggregation (Ellis, 2001). To observe other intracellular interactions, organelle sizes were normalised and plotted in relation to the normalised cell area (Fig. 7B), as well as the sum of organelles sizes (Fig. S6C,D); thus, their respective adaptations became clearer. The change in size of organelles could be matched up in pairs: cell-nucleus, vacuole-MVB and mitochondria-LD, suggesting complementary or co-dependent functions in the heat-shock response. After the 15 min timepoint, both the cell and the nucleus have the same rate of increase in size, supporting a tight link between the two structures, as previously also shown in fission yeast (Neumann and Nurse, 2007). Vacuoles and MVBs both rapidly increase in size within the first 15 min and show similar rates of change in size throughout the rest of the time course. A possible mechanism for their connection is iterated in the Discussion. Mitochondria and LDs also have similar rates of increase in size starting the 15 min timepoint, revealing dynamic interaction between the two organelles. Indeed, LDs have previously been observed to serve as an energy source for mitochondria during nutrient stress (Rambold et al., 2015). In summary, this approach revealed a rapid, complex and organelle-specific structural response to heat shock.

## DISCUSSION

**Fig. 8.**
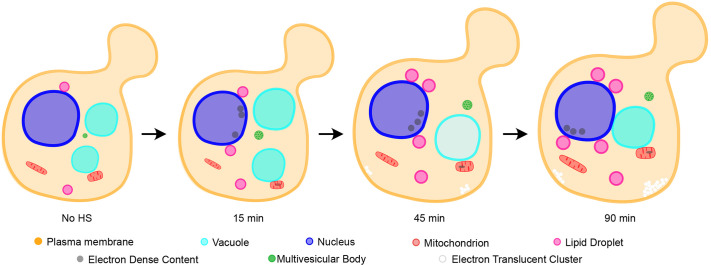
**Model of structural changes occurring during mild heat shock.** Model of structural changes occurring during a 90 min mild heat shock.

Our large-scale electron microscopy approach allowed us to both analyse and quantify cellular changes during heat shock in a broad perspective and at high resolution. This showed that mild heat shock affects the size and form of every organelle we studied (Fig. 8), some more severely than others, and some with no previously known roles during heat shock, such as the MVB. It also allowed us to describe a previously undescribed cellular structure – ETCs.

### ETCs are triggered by heat stress

To our knowledge there are no previous descriptions of ETCs in the literature, although similar structures are clearly visible in previously published electron micrographs of temperature-sensitive mutants at restrictive temperature (Novick et al., 1980; Poon et al., 1999; Marini et al., 2020). Here, we can only speculate as to their nature and function: the electron translucency of ETCs is similar to that of LDs, indicating that they potentially are lipid deposits. However, the difference in morphology and the lack of BODIPY staining at the cell periphery suggests that the ETCs are not composed of the same neutral lipids that are found in LDs (Hsieh et al., 2012). By contrast, other membraneless structures that are known to accumulate during stress, such as p-bodies and stress granules, would be visible in electron micrographs as electron-dense structures as they contain mRNA that is stained dark by most electron microscopy preparation protocols (Cougot et al., 2012).

As opposed to other cellular components, throughout the heat shock time course, the area occupied by ETCs continued to steeply increase, accumulating near the plasma membrane. A possible explanation for this localisation could be related to the fluidity of the membrane, which is known to increase under stress, especially heat stress, in various organisms (Dynlacht and Fox, 1992; Lloyd et al., 1993; Mejía et al., 1995; Török et al., 2014) and has been suggested to initiate the heat-shock protein response (Balogh et al., 2005). A more fluid membrane, which has been observed to correlate with higher permeability (Wilkes et al., 1989), could allow material, such as extracellular fluid or components of the cell wall, to be released into the cytoplasm and observed as ETCs in electron micrographs. Furthermore, unsaturated lipids might be deposited nearby to regulate membrane rigidity at higher temperatures. Alternatively, it is also possible that the ETC could be an accumulation of carbohydrates, as it has been shown that the sugar trehalose accumulates in cells as a cytoprotectant during stress (Ribeiro et al., 1994; de Virgilio et al., 1994).

### MVBs are involved in the heat-shock response of the cell

MVBs are involved in the transport of membranous and cytoplasmic content but have no documented roles in the heat-shock response to date. Although the number of MVBs remains constant throughout heat shock, their size increases significantly. This could mean that, although their synthesis is not affected, the activity of existing MVBs is nevertheless upregulated. Because intraluminal vesicles formed from the MVB membrane do not only internalise membrane and membrane proteins, but also cytoplasmic content, they offer a proteasome-independent degradation of both membrane- and cytosolic proteins. Thus, their large increase in size throughout heat shock could indicate a need to support the degradation and sorting machinery of the cell as MVBs transport ubiquitylated cargo (Piper and Katzmann, 2010). Interestingly, the increase in size of MVBs and of vacuoles follows a similar path (Fig. 7A,B), supporting their potential influence on each other's activity.

### Vacuolar morphology is affected by heat stress

Beside an increase in vacuole size of almost 50% after heat stress, one of the most immediately noticeable changes is the difference in their morphology. Owing to the nature of the preparation protocol, all sets show fluctuations in the electron density of vacuoles; however, set 3 is an outlier in its overall high numbers of electron-dense vacuoles. Our results suggest that the different vacuolar electron densities reflect its internal pH and that the uranyl acetate stain can act as a potential pH probe in electron micrographs.

We see that heat stress affects the vacuolar electron density in a similar manner to ageing. In electron micrographs of dividing cells, the daughter had either higher or equal vacuolar electron density when compared with its mother. Previous research has shown that the pH of the mother vacuole is already increased after one division (Hughes and Gottschling, 2012). It may seem that deacidification of the vacuole during heat stress stems from an export of protons to the cytoplasm to cause the acidification necessary for the induction of heat shock factor 1 (HSF1), the main regulator gene of the heat-shock response. However, it has been suggested that cells instead depend on the import of extracellular protons for acidification required to induce HSF1 (Triandafillou et al., 2020). Observations of vacuolar invaginations, indentation and cytoplasmic vesicles (Fig. S1 and Ishii et al., 2018) may indicate indirect support of cytoplasmic acidification by increasing the proportion of imported protons relative to cytoplasmic content. The cause and mechanism for vacuolar deacidification remain unclear but the resulting impairment in function (Hughes and Gottschling, 2012) offers a potential explanation for the increase in the size of MVBs due to inhibition of trafficking to the vacuole.

Because we show here that UA staining has the potential to be used as a pH indicator in cells, it is important to rule out the possibility that UA staining of vacuoles is only influenced by their biomolecular content. UA binds to phosphate and carboxyl groups of molecules (Hayat, 2000). Therefore, it normally stains proteins and nucleic acids in cells (Reynolds, 1963; Hawes et al., 2007). Accordingly, we used two different mutants: *prb1*Δ, which has more acidic vacuoles than wild-type cells; and *vma2*Δ, which has deacidified vacuoles when compared with wild type. The acidic vacuoles of *prb1*Δ were more electron dense (Fig. 3E, Fig. S1B), whereas the more basic vacuoles of the *vma2*Δ mutant were electron translucent (Fig. 3E). Both of these mutants have inhibited proteolytic activity (Nakamura et al., 1997; Zubenko and Jones, 1981), which should cause their vacuoles to have a higher biomolecular content. If the presence of biomolecules such as proteins and nucleic acids alone influenced the vacuolar staining, these mutants would not have exhibited such different staining patterns, which supports our hypothesis that UA preferentially stains acidic environments.

### Vacuolar membrane contact sites with the nucleus increase in size

Contact sites between the nucleus and vacuoles increase in size throughout heat shock, and the first three time points differ significantly from the 90 min time point. It is also during the first three time points that the increase in length of the MCS is proportional to the size of the respective organelles (Fig. S5A), suggesting that initially nuclear-vacuolar interaction is simply sustained. Starting at 30 min, and until the end of the time course, the membrane contact sites start becoming longer than expected, where the expected value is calculated based on the circumference of the organelles forming the contact site (see Materials and Methods). Owing to the large size of vacuoles in comparison with the observed microscopy sections, contact sites with vacuoles may be under-represented in this quantification. Nuclear vacuolar junctions have been shown to be sites of piecemeal microautophagy of the nucleus (Roberts et al., 2003). An increase in the size of the MCS and vacuoles, as well as the presence of EDC in the nucleus, suggest that this process is increased during heat shock. Furthermore, nuclear vacuolar junctions are involved in lipid metabolism (Kohlwein et al., 2001; Levine and Munro, 2001), which is in line with our observations of enlarged and more numerous lipid droplets being found in the cell after heat shock and overall longer MCSs.

Similarly, despite an increase in size of both vacuoles and mitochondria, contact sites between the organelles do not increase in size or number, as may be expected. However, at 45 min of heat shock, when vacuoles are largest in size, MCSs between the vacuole and the mitochondria are shorter than the expected value (Fig. S5B). As vacuoles and mitochondria are closely linked, and vacuolar deacidification contributes to mitochondrial deterioration (Hughes and Gottschling, 2012), it is possible that keeping the number and size of contact sites constant prevents excessive damage to the cell during heat stress. Overall, this investigation shows that there is a specificity in the regulation of organelle contact sites during heat shock.

### A holistic approach relevant across multiple fields of research

It is valuable to track global cellular changes occurring during heat shock to better understand how the stress response is coordinated. It is also important to be aware of these global changes when, for example, working with temperature-sensitive mutants where phenotypes attributed to the altered ts allele may instead be a direct or indirect effect of the heat shock itself.

Overall, this study has revealed how cell morphology is influenced by heat shock. Changes in structure, number and size of organelles is related to the molecular activity behind those processes and their careful and balanced interplay. This quantification of organelle changes highlights the importance of a holistic approach to answering research questions, and creates a map and reference for those interested in reaction and adaptation to stress by eukaryotic cells.

## MATERIALS AND METHODS

### Yeast strains

Yeast strains used in this study are derivatives of BY4741, here referred to as wild type. Deletion strains have the gene of interest replaced by the KanMX cassette (EUROSCARF). *HSP104-GF* and *VPH1-GFP* are from the GFP-tagged collection (Huh et al., 2003). Strains are listed in Table S1.

### Growth conditions

Cells were cultured at 30°C in rich YPD medium to mid-exponential phase. For heat shock, cell cultures were shifted to 38°C for indicated times before further analysis. The 0 min time point refers to the cultures at 30°C, immediately before being exposed to heat shock.

### Fluorescence microscopy

For vacuolar pH analysis, 1 OD_600_ of logarithmically growing cells were washed once in YPD+100 mM HEPES (pH 7.6) and stained with 50 µM BCECF-AM (ThermoFisher) dissolved in the same buffer at 30°C for 30 min. Cells were washed twice in pre-warmed 100 mM HEPES (pH 7.6)+2% glucose, resuspended in pre-warmed YPD and allowed to grow at either 30°C or 38°C for 45 min. Cells were washed in pre-warmed 100 mM HEPES (pH 7.6) +2% glucose then immediately imaged. For quinacrine staining, 1 OD_600_ of logarithmically growing cells were washed once in YPD+100 mM HEPES (pH 7.6) and incubated in 100 µl 200 µM quinacrine (Sigma-Aldrich) dissolved in YPD+100 mM HEPES (pH 7.6) for 10 min at 30°C. Cells were kept on ice for 5 min, washed twice in ice-cold 100 mM HEPES (pH 7.6) +2% glucose, and kept on ice until imaged.

For visualisation of lipid droplets, ∼1 OD_600_ of wild-type cells were harvested by centrifugation, resuspended in 1 ml PBS and incubated with 1 µg/ml BODIPY 493/503 (ThermoFisher) diluted in DMSO for 15 min. Cells were washed once in PBS before imaged. Hsp104-GFP- and Vph1-GFP-expressing cells were grown logarithmically, collected by centrifugation, and imaged directly.

Imaging of live yeast cells was performed using a Zeiss Axio Observer Z1 inverted fluorescent microscope equipped with an AxioCam MRm camera (Zeiss). A Plan Apo 100× oil objective NA:1.4 with the filter set to 38 HEeGFP was used.

### Electron microscopy

Yeast cultures were grown to an OD_600_ of 0.5, control cells were kept at 30°C and treated cells were heat shocked at 38°C. After removal of medium by filtering through a 0.22 µm filter (Ding et al., 1993; Winey et al., 1995; O'Toole et al., 1997; Müller-Reichert et al., 2003), the cells were scraped off the filter membrane with a toothpick, transferred to an aluminium carrier and high-pressure frozen in a Wohlwend Compact 3. Freeze substitution was performed in a Leica AFS2 with incubation in 2% uranyl acetate in acetone (UA; SPI Supplies) for 1 h (Hawes et al., 2007), rinsing in acetone and step-wise (steps of 20%, 40%, 50%, 80% and 3×100%) embedding in Lowicryl HM20 resin (Polysciences) at −50°C, followed by a 5 day polymerisation under UV light while allowing the samples to reach room temperature. Lowicryl HM20 resin allows the clear visibility of morphology (Höög et al., 2007, 2013) and potentially necessary immuno-gold labelling (Hawes et al., 2007). Polymerised resin blocks were sectioned at 70 nm using a Reichert Ultracut S and placed on Formvar-coated 200-mesh or slot copper grids. Grids were contrast stained using a 2% aqueous UA solution for 5 min and Reynold's lead citrate (Reynolds, 1963) for 1 min. Samples were imaged at 120 kV on a FEI Tecnai G2 Spirit with an FEI Ceta 16 M camera (4k×4k) (Thermo Fisher Scientific) and a pixel size of 1.1 nm for a magnification of 9300×.

### Image analysis and quantification

Sections from the central part of the cell (large diameter) that contained organelles were imaged from only the central section in a serial section ribbon, to ensure that no cells were imaged in duplicate. In total, 2143 micrographs of cells were analysed for the time course, corresponding to over 350 cells per timepoint, on average. Electron micrographs were quantified using the program IMOD (Kremer et al., 1996), by drawing outlines of the structures of interest. Structures such as vacuoles, mitochondria and nuclei were also classified by eye according to morphology. The electron density of vacuoles was analysed visually by the first author (K.S.K.) in relation to the electron density of the cytoplasm. The length of contact sites was put in relation to the circumference of the respective organelles in contact.

Owing to the large size of vacuoles, their number per cell was determined in fluorescence microscopy images of Vph1-GFP (Manolson et al., 1992) cells by manually counting ≥200 cells per replicate and time point in Fiji (Schindelin et al., 2012). Vacuolar area was quantified for ≥200 vacuoles per replicate and time point using the freehand area tool in Fiji on maximum projections of microscopy images of Vph1-GFP cells. Vacuolar acidity was quantified using Fiji by measuring BCECF-AM mean fluorescence intensity of ≥30 vacuoles per condition from three independent experiments.

The contact site fraction of a MCS, *CSF*, represents the fraction of the total adjacent organelle circumference that is a MCS and was calculated as follows for time points *t*:
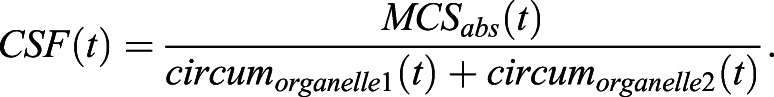


At the beginning of the heat-shock time course (*t*=0), the ratio of the respective median values is *CSF*_0_ and can be used as indicator for the expected length of a MCS, using the respective median values:
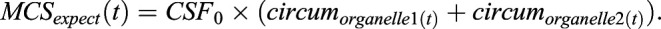


### Statistical analysis

After the measurements were obtained from the models, replicates 1, 2 and 3 were treated as one batch. Normality for each timepoint and organelle was checked using a Shapiro–Wilk test. Where possible (cell and MVB area), measurement distribution was normalised using a Box–Cox transform (Box and Cox, 1964) (λ=0.63 and λ=0.4), equal variances confirmed with a Bartlett's test and one-way ANOVA performed (cell area). In the case of unequal variances, pairwise *t*-tests using non-pooled standard deviation were performed (MVB area). Pairwise group comparisons were performed using the R package *emmmeans* (https://cran.r-project.org/package=emmeans). Non-normally distributed data that could not be normalised were analysed with Dunn's test. For numbers of organelles, pairwise *t*-tests with non-pooled standard deviation were used. All *P*-values were adjusted according to Holm (1979) and are shown in Table S2.

Where significant differences between replicates 1, 2, and 3 were found, sets were analysed individually and the most conservative *P*-values are shown (these instances are mitochondrial and LD area). Scatter plots show non-transformed data to allow more intuitive interpretation of area values.

## Supplementary Material

Supplementary information

Reviewer comments
