# Supplementary material for: Large organellar changes occur during mild heat shock in yeast
Source: J Cell Sci. 2021 Aug 11;135(5):jcs258325. doi: 10.1242/jcs.258325 (PMC8403982; doi:10.1242/jcs.258325)
Supplement: Supplementary information [file joces-135-258325-s1.pdf]

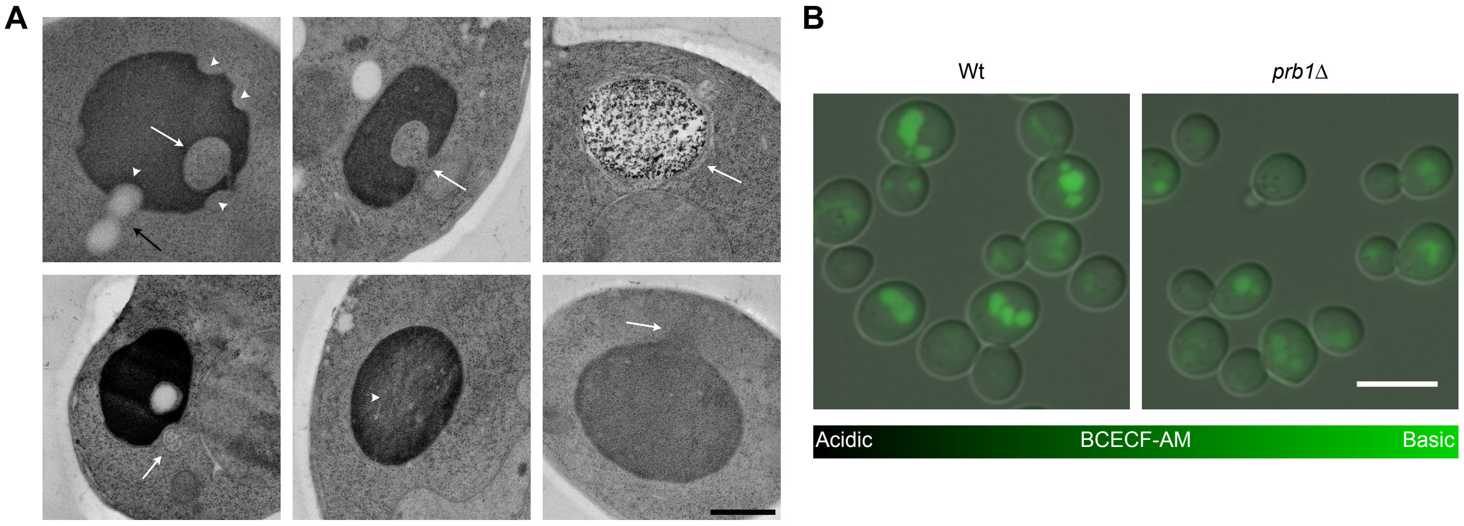

Fig. S1. Vacuoles have various altered morphologies and pH A) Gallery of vacuolar morphologies in heat shocked cells (Top) Left: indentations in the vacuolar membrane (arrowheads), LDs (black arrow) and cytoplasmic vesicle contained inside the vacuole (white arrow), Middle: Invagination into vacuole (arrow), Right: Dark precipitates deposited onto the vacuole (arrow). (Bottom) Left: an MVB (arrow) near an indentation of a vacuole and an LD enclosed by the vacuolar lumen, Middle: electron dense ring at the periphery of the vacuole (arrowhead), Right: vacuole seemingly "leaking" content into cytoplasm (arrow). Scale bar 500 nm B) Fluorescence microscopy of indicated strains. The fluorescent pH-sensitive probe BCECF accumulates in yeast vacuoles and shows vacuolar pH. The more fluorescence, the more basic vacuolar pH. Scale bar 5µm

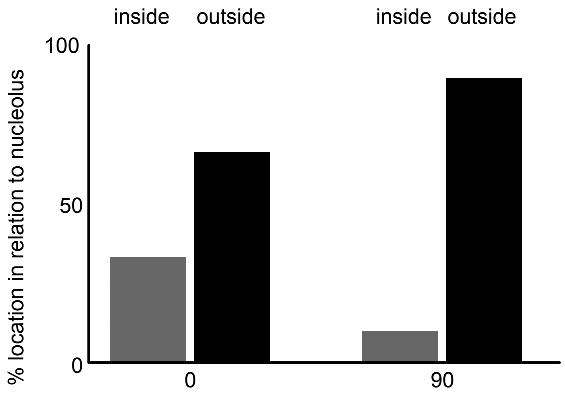

Fig. S2. EDC is mostly found outside of the nucleolus Proportion of nuclei that contain EDC inside or outside the nucleolus. n=12 and 134 nuclei at 0 and 90 min heat shock respectively.

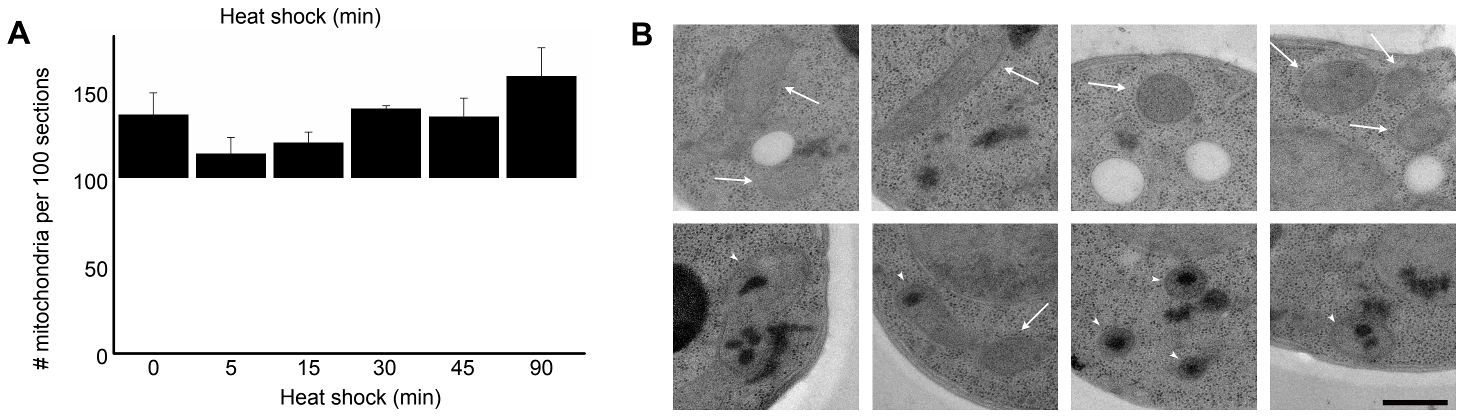

Fig. S3. Number of mitochondria does not vary significantly throughout heat shock when observed with EM A) Number (mean±s.e.m.) of mitochondria per 100 cell sections in cells subjected to indicated times of HS. 300+ cells were analyzed per time point. B) Gallery of mitochondria without EDC (top row, arrows) and with EDC (bottom row, arrowheads). Scale bar 500 nm

Journal of Cell Science • Supplementary information

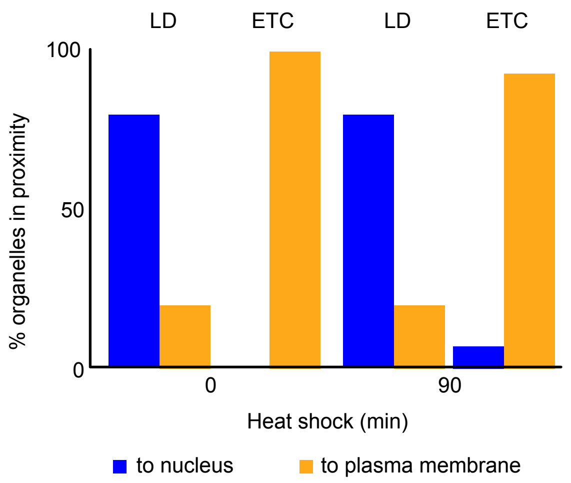

Fig. S4. Lipid droplets are closer to the nucleus and electron translucent clusters are closer to the plasma membrane Proportion of lipid droplets (LD) and electron-translucent clusters (ETC) closer to the nucleus or plasma membrane before heat shock and after 90 min heat shock. n=50 LD and 22 ETC at 0 min and n=50 LD and 57 ETC at 90 min heat shock.

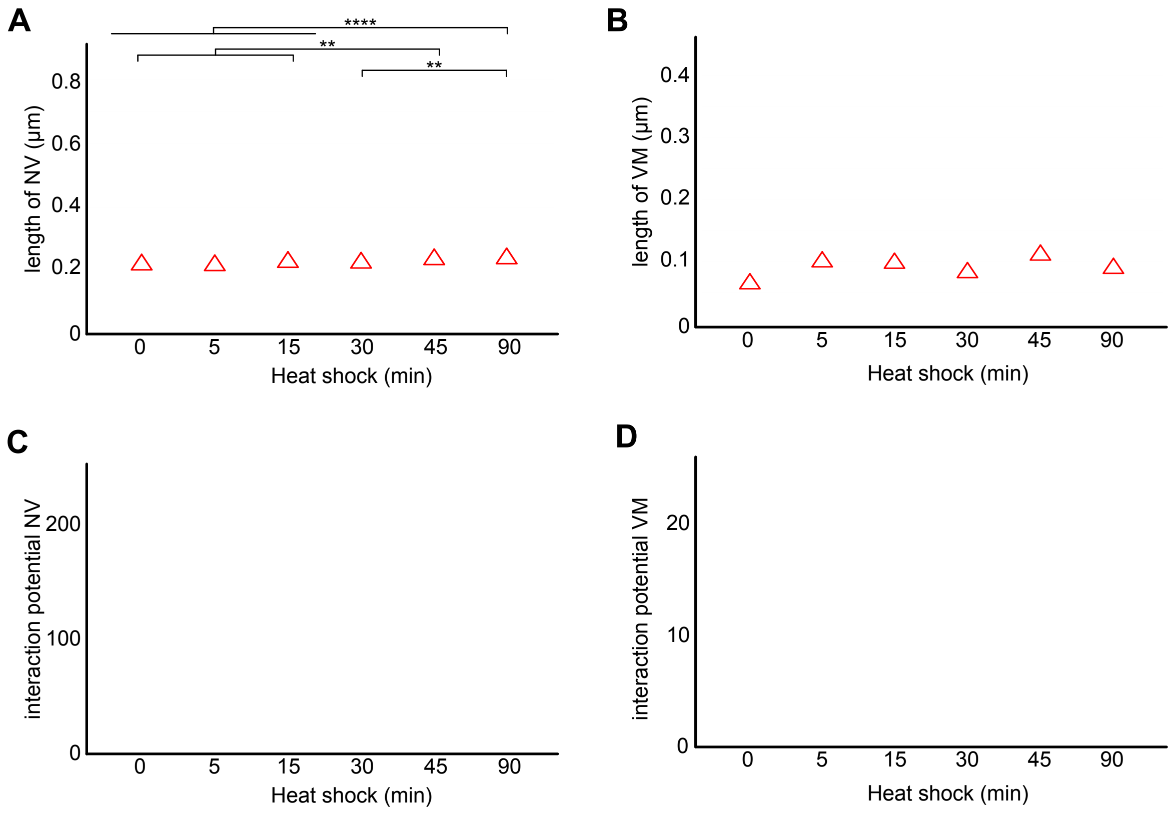

Fig. S5. Membrane contact sites are influenced by heat shock A) Absolute length of contact site between nucleus and vacuole (NV). Black line is the median and error bars the interquartile range, red triangles are the expected values scaled to size of adjacent organelles. Significances: (**) $p \leq 0.01$, (****) ≤ 0.0001. B) Absolute length of contact site between vacuole and mitochondria (VM). Black line is the median and error bars the interquartile range. No significant differences found. C) Interaction potential (arbitrary units) between the nucleus and vacuole, corresponding to the average proportion of sections with contact sites multiplied by the average absolute length in nm. D) Interaction potential (arbitrary units) between the vacuole and mitochondria, corresponding to the average proportion of sections with contact sites multiplied by the average absolute length in nm. Error bars are s.e.m.

Journal of Cell Science • Supplementary information

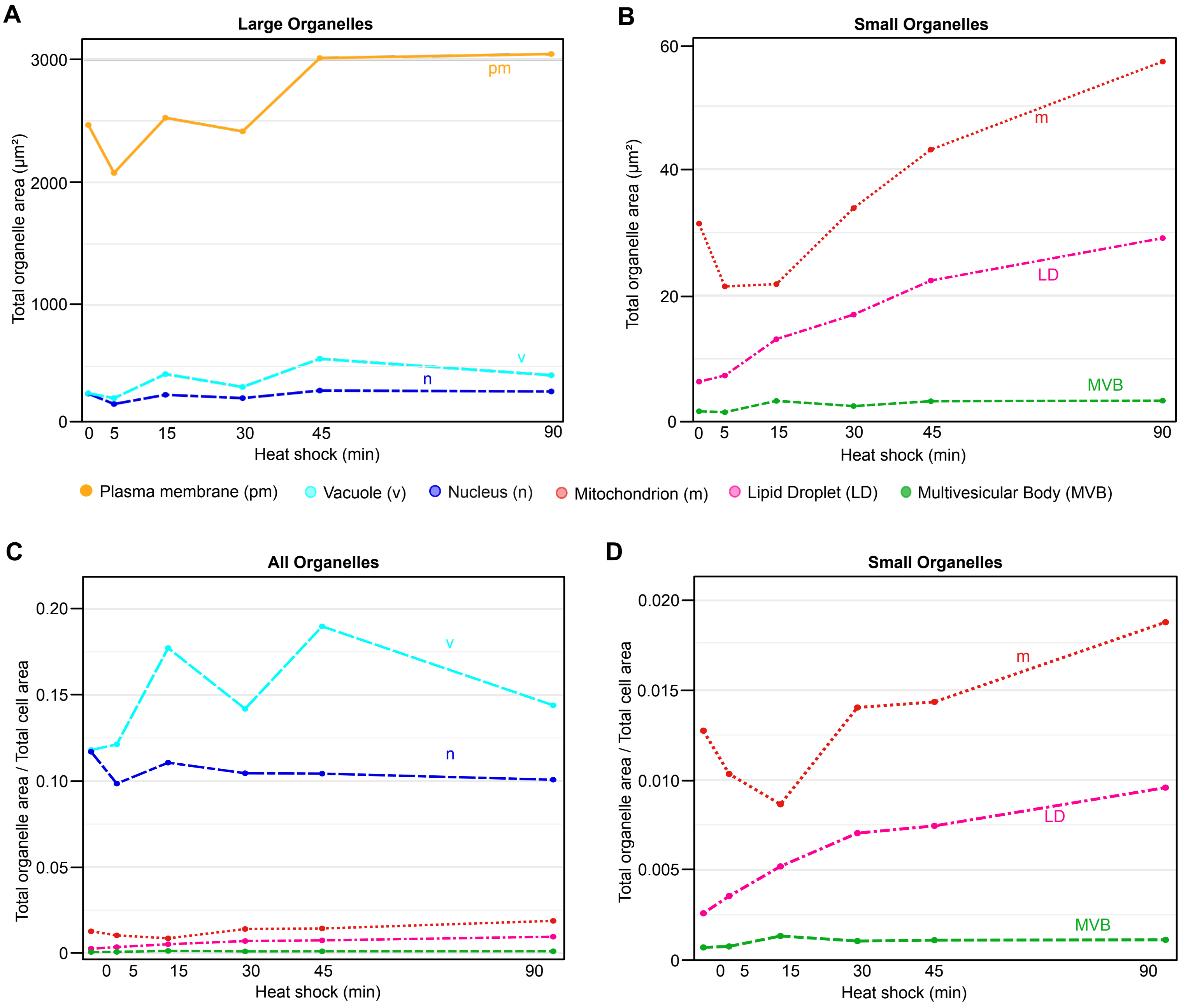

Fig. S6. Total sum of organelle sizes A) and B) Sum of the total organelle area of larger (A) and smaller (B) organelles. C) and D) Ratio of the total sum of organelle area divided by the total sum of cell area. D) Zoom in on graph C) for smaller organelles.

Journal of Cell Science • Supplementary information

Table S1. Strains used in this study

Strain	Genotype	Background/source
WT	*MAT**a** his3Δ1 leu2Δ0 met15Δ0 ura3Δ0*	BY4741; EUROSCARF
Hsp104-GFP	*MAT**a** his3Δ1 leu2Δ0 met15Δ0 ura3Δ0 HSP104-GFP-HIS3*	BY4741; Invitrogen
Vph1-GFP	*MAT**a** his3Δ1 leu2Δ0 met15Δ0 ura3Δ0 VPH1-GFP-HIS3*	BY4741; Invitrogen
vma2Δ	*MAT**a** vma2Δ::kanMX4 his3Δ1 leu2Δ0 met15Δ0 ura3Δ0*	BY4741; YKO collection. EUROSCARF
prb1Δ	*MAT**a** prb1Δ::kanMX4 his3Δ1 leu2Δ0 met15Δ0 ura3Δ0*	BY4741; YKO collection. EUROSCARF

Table S2. P-value tables

Figure 2B

time point	0	5	15	30	45	90
5	0.4485	-	-	-	-	-
15	0.9979	0.7184	-	-	-	-
30	0.0235	0.0001	0.005	-	-	-
45	0.0001	0.0001	0.0001	0.0741	-	-
90	0.0001	0.0001	0.0001	0.0001	0.3792	-

Figure 2C

time point	0	5	15	30	45	90
5	0.0142	-	-	-	-	-
15	0	0.0118	-	-	-	-
30	0	0.0128	0.4993	-	-	-
45	0	0	0.0196	0.0221	-	-
90	0	0.0025	0.8718	0.596	0.686	-

Figure 4B

time point	0	5	15	30	45	90
5	0.634	-	-	-	-	-
15	0.0192	0.2843	-	-	-	-
30	0.7846	0.4435	0.2746	-	-	-
45	0.5628	0.5384	0.0021	0.4499	-	-
90	0.548	0.1952	0.0001	0.1007	0.7794	-

Figure 4E

time point	0	5	15	30	45	90
5	0.3524	-	-	-	-	-
15	0.1435	0.6828	-	-	-	-
30	0.4958	0.0005	0.0012	-	-	-
45	0.0399	0.1054	0	0.6376	-	-
90	0.0115	0.1412	0	0.347	0.8467	-

Figure 5B

time point	0	5	15	30	45	90
5	1	-	-	-	-	-
15	0.19	0.19	-	-	-	-
30	1	1	1	-	-	-
45	1	1	1	1	-	-
90	1	1	1	1	1	-

Figure 5C

time point	0	5	15	30	45	90
5	0.3207	-	-	-	-	-
15	1	0.9458	-	-	-	-
30	0.0021	0.6915	0.0273	-	-	-
45	0.00047	0.4063	0.00065	1	-	-
90	0.000089	0.0206	0.000067	1	1	-

Figure 5E

time point	0	5	15	30	45	90
5	1	-	-	-	-	-
15	0.892	0.371	-	-	-	-
30	0.900	0.371	1	-	-	-
45	0.371	0.102	1	0.890	-	-
90	0.227	0.035	0.892	0.371	1	-

Figure 5F

time point	0	5	15	30	45	90
5	0.861	-	-	-	-	-
15	2.10E-08	2.40E-11	-	-	-	-
30	6.30E-06	9.90E-08	9.86E-01	-	-	-
45	5.20E-12	2.00E-14	6.90E-02	1.50E-01	-	-
90	3.90E-10	8.30E-13	8.59E-01	8.61E-01	8.61E-01	-

Figure 6C

time point	0	5	15	30	45	90
5	0.7323	-	-	-	-	-
15	0.6229	0.3876	-	-	-	-
30	0.1076	0.3306	0.1512	-	-	-
45	0.0115	0.0768	0.0114	0.7592	-	-
90	0.0000	0.0002	0.0000	0.0265	0.1313	-

Figure 6F

time point	0	5	15	30	45	90
5	1.0000	-	-	-	-	-
15	0.7586	0.4038	-	-	-	-
30	1.0000	0.9281	1.0000	-	-	-
45	0.0273	0.4259	0.1597	0.0023	-	-
90	0.7002	0.8885	0.9291	0.1631	0.6219	-

Figure S5A

time point	0	5	15	30	45	90
5	0.4455	-	-	-	-	-
15	0.5090	0.4566	-	-	-	-
30	0.0753	0.2922	0.2880	-	-	-
45	0.0008	0.0112	0.0043	0.2670	-	-
90	0.0000	0.0000	0.0000	0.0052	0.2191	-

Journal of Cell Science • Supplementary information
