# Supplementary material for: Large organellar changes occur during mild heat shock in yeast
Source: J Cell Sci. 2021 Aug 11;135(5):jcs258325. doi: 10.1242/jcs.258325 (PMC8403982; doi:10.1242/jcs.258325)
Supplement: Reviewer comments [file joces-135-258325_review_history.pdf]

Large organellar changes occur during mild heat shock in yeast

Katharina S. Keuenhof, Lisa Larsson Berglund, Sandra Malmgren Hill, Kara L. Schneider, Per O. Widlund, Thomas Nyström and Johanna L. Höög

DOI: 10.1242/jcs.258325

Editor: James Olzmann

Review timeline
Original submission: 25 January 2021
Editorial decision: 1 March 2021
First revision received: 31 May 2021
Accepted: 25 June 2021

Original submission

First decision letter

MS ID#: JOCES/2020/258325

MS TITLE: Large Organellar Changes occur during Mild Heat Shock in Yeast

AUTHORS: Katharina Keuenhof, Lisa Larsson Berglund, Kara L Schneider, Sandra Malmgren Hill, Per O Widlund, Thomas Nyström, and Johanna L Höög
ARTICLE TYPE: Research Article

We have now reached a decision on the above manuscript.

To see the reviewers' reports and a copy of this decision letter, please go to: https://submit-jcs.biologists.org and click on the 'Manuscripts with Decisions' queue in the Author Area. (Corresponding author only has access to reviews.)

As you will see, the reviewers gave favourable reports but raised some critical points that will require amendments to your manuscript. The comments from Reviewer 1 regarding the details of how measurements were made and organelle area determined for 3-dimensional organelles, are particularly important to address.

We are aware that you may be experiencing disruption to the normal running of your lab that makes experimental revisions challenging. If it would be helpful, we encourage you to contact us to discuss your revision in greater detail. Please send us a point-by-point response indicating where you are able to address concerns raised (either experimentally or by changes to the text) and where you will not be able to do so within the normal timeframe of a revision. We will then provide further guidance. Please also note that we are happy to extend revision timeframes as necessary.

Please ensure that you clearly highlight all changes made in the revised manuscript. Please avoid using 'Tracked changes' in Word files as these are lost in PDF conversion.

I should be grateful if you would also provide a point-by-point response detailing how you have dealt with the points raised by the reviewers in the 'Response to Reviewers' box. Please attend to all of the reviewers' comments. If you do not agree with any of their criticisms or suggestions please explain clearly why this is so.

© 2021. Published by The Company of Biologists under the terms of the Creative Commons Attribution License (https://creativecommons.org/licenses/by/4.0/).

Reviewer 1

Advance summary and potential significance to field

In this manuscript, the authors utilize electron microscopy to characterize structural changes in budding yeast cells during heat shock. Among their main findings:
- Cell and organelle have characteristic size changes with respect to time after shock.
- Vacuoles undergo size expansion and pH changes.
- Nucleus and vacuole contacts increase.
- Multi-vesicular bodies and lipid droplets proliferate in number.

I agree that changes to cellular structure in response to stress is of interest to field. Many of the authors findings are intriguing, including the change in electron density within different organelles.

Comments for the author

There are a number of fundamental concerns on the manuscript's experimental and analytical methods that need addressing to ensure suitability for publication.

1. The authors emphasize that a great number of cell sections were imaged and analyzed by EM, but it was unclear how many cells this actually corresponds to.
Pg 19 states that "…2143 micrographs were analysed for the time course corresponding to a full-cell volume of 37 cells…" Our interpretation is that 37 individual cells were analyzed. If this is the case, it seems like a small number, especially if those 37 cells are taken from 6 timepoints, roughly averaging 6 cells per timepoint.

The small number of individual cells also calls into question other analysis performed. For example, Pg 5 and Fig 1B report that 2143 plasma membranes were measured. Similar numbers are reported for other organelles. We interpret this to indicate that the authors have taken measurements of multiple sections of the same individual cells/organelles, then treated these as independent measurements for statistical analysis, despite the fact they will be highly correlated. The authors need to clarify, did these 2143 measurements come from 2143 independent cells, or 37? If the latter, this would seem like a major flaw for the author's analysis, and they would need to reperform their analysis with only one measurement of size per cell/organelle.

2. It is unclear how cell and organelle size were determined from EM cross-sections. For sphere-like 3-D objects, the cross-sectional area will be highly dependent on whether the section is taken closer to the object's equator or to its poles. Is there a method for choosing which cross-section is representative of the overall object? If so, this should be added to the text. If not, this would reflect a major weakness with the image analysis. In a related question the authors present analyses in Fig 7 comparing organelle to cell size. This seems to assume that the same cross-section provides an accurate relative measurement for both the organelle and cell, but this would be highly dependent on how the cell and organelle are arranged. Again, the authors should clarify how cell and organelle size ratios were derived.

3. Given that many organelles consist of multiple individuals (i.e. vacuole and mitochondria), reporting only on the average individual size of these organelles seems insufficient, and that a total size of all of the same type of organelle should also be analyzed. Unfortunately, if a single cell has multiple copies of the same organelle, it's unclear how to create a combined size from measurements of cross-sectional area.

Additional minor comments:

4. The interpretation of the change in electron density in the vacuole as relating to acidification was interesting, but seemed under-supported. The authors provide a correlative argument that electron density matches the intensity of the BCECF dye in fluorescence experiments, and from this assert a connection. But the mechanism of this seems highly speculative. Is there other evidence or a reference that uranyl acetate staining depends on pH? It seems possible that the change in electron density would signify a different in the protein/nucleic acid/lipid concentration in the

© 2021. Published by The Company of Biologists under the terms of the Creative Commons Attribution License (https://creativecommons.org/licenses/by/4.0/).

vacuole, have the author's ruled out this possibility? Further testing of this possibility would be helpful.

5. The authors conclude that nucleus-vacuole contacts are increased, based on the relatively large increase in N-V contact length beyond what is expected compared to the size increases observed for the vacuole. However, it's not readily apparent what the expected change should be here. Do the authors have a geometric model to explain their expectation? Is the expectation based on the percent increase in the vacuole cross-sectional area? How is the change in cell size incorporated into this estimate? This would seem to provide a major constraint for organelle organization.

6. The authors show the rise of electron dense structures in the nucleus/mitochondria, and electro translucent structures in the cytoplasm.
Further discussion on these might be useful, in particular, are the EDC's in the nucleus related to the nucleolus?

7. It's unclear why in different statistical analyses, it seems like multiple timepoints were grouped together (examples, Fig 2B, 2C, 4B, 4E, 6C.)
Furthermore, the groupings are inconsistent, and the data for some timepoints seems to be used in multiple statistical comparisons, which seems problematic.
Please provide an explanation for how data for different timepoints were determined to be grouped together for statistical analysis.

<u>Reviewer 2</u>

Advance summary and potential significance to field

This is the first systematik ultrastructural analysis of cells in response to mild heat shock. This data can/will serve as a basis for further functional and structural studies.

Comments for the author

In this paper, the authors have analyzed ultrastructural changes in budding yeast in response to mild heat shock. This is a nice systematic study on this topic, and I have only some minor comments:

1. Introduction

The authors mention the importance of the heat shock phenomenon in neurodegenerative human diseases.
Why is it then useful to study the cytological changes by using yeast as a model? What is the motivation here to use budding yeast? This aspect should be introduced to the interested reader.

2. Results

This comment is also related to the section Materials & Methods. Cells were "randomly" chosen. I think the procedure should be explained in more details. Did the authors, for instance, cut serial sections? If so, how did the authors exclude multiple countings of the same cell?
In Figure 1B, the lower mid image is rather dark, and it is hard to see the cytological details. Another image should be selected.
In general, it should be avoided to put letters directly on the marked organelles (for instance the 'm' for the mitochondria directly on the organelle). This way, information is hidden for the reader.
As for figure 2B, I am not sure about the presentation of the data. The authors should think about presenting the average of all three experiments. The distribution could be given in a suppl. figure.
I'm also not sure about the grouping of times 0-15 and 30-90 min for the statistical analysis and testing. What is the rationale behind this grouping?
As for figure 3A, has the density of the vacuoles been evaluated/grouped by 'eye'?
For Fig 4D, could the authors show more examples of the mitochondria in a suppl. figure?

© 2021. Published by The Company of Biologists under the terms of the Creative Commons Attribution License (https://creativecommons.org/licenses/by/4.0/).

3. Discussion

Personally, I think Figure 7A-B should be shifted to the results section. I think this part of the figure is misplaced. Fig. 7C could stay in the discussion, as a kind of summary.
The matching up in pairs is a very interesting result. The authors should comment on this. What could be the biological message here? I would like to hear the opinion of the authors on this.

The first sentence of the discussion: change ‘allows’ to ‘allowed’.

4. Materials & methods

I think the part about the sample preparation for electron microscopy should be expanded. For the protocol on yeast filtration, the original paper for this procedure should be cited. As for the use of the resin, what are the details for infiltration with HM20 and the polymerization of the resin? How was this done? Why did the authors use this specific resin for their experiments? I guess, the clear visibility of the membranes in resin was the argument here.

As for the selection of the cells for quantification, see my comment above.

With these minor corrections, this paper should be accepted for publication in the Journal of Cell Science.

First revision

Author response to reviewers' comments

Point-by-point response to the reviewers

We wish to thank the reviewers of the manuscript for their comments and suggestions. In our reply below we acknowledge and address all the points that were raised. We appreciate that all the proposed experiments and clarifications improved the quality of our work.

Reviewer 1

Reviewer 1 Advance Summary and Potential Significance to Field:

“In this manuscript, the authors utilize electron microscopy to characterize structural changes in budding yeast cells during heat shock. Among their mainfindings:
- Cell and organelle have characteristic size changes with respect to time after shock.
- Vacuoles undergo size expansion and pH changes.
- Nucleus and vacuole contacts increase.
- Multi-vesicular bodies and lipid droplets proliferate in number.

I agree that changes to cellular structure in response to stress is of interest to field. Many of the authors findings are intriguing, including the change inelectron density within different organelles.”

Reviewer 1 Comments for the Author:

“There are a number of fundamental concerns on the manuscript’s experimental and analytical methods that need addressing to ensure suitability for publication.”

1. “The authors emphasize that a great number of cell sections were imaged and analyzed by EM, but it was unclear how many cells this actually corresponds to. Pg 19 states that “…2143 micrographs were analysed for the time course, corresponding to a full-cell volume of 37 cells…” Our interpretation is that 37 individual cells were analyzed. If this is the case, it seems like a small number, especially if those 37 cells are taken from 6 timepoints, roughly

© 2021. Published by The Company of Biologists under the terms of the Creative Commons Attribution License (https://creativecommons.org/licenses/by/4.0/).

averaging 6 cells per timepoint. The small number of individual cells also calls into question other analysis performed. For example, Pg 5 and Fig 1B report that 2143 plasma membranes were measured. Similar numbers are reported for other organelles. We interpret this to indicate that the authors have taken measurements of multiple sections of the same individual cells/organelles, then treated these as independent measurements for statistical analysis, despite the fact they will be highly correlated. The authors need to clarify, did these 2143 measurements come from 2143 independent cells, or 37? If the latter, this would seem like a major flaw for the author's analysis, and they would need to reperform their analysis with only one measurement of size per cell/organelle."

Thank you for pointing out this ambiguity in the manuscript. Indeed, the 2143 micrographs correspond to 2143 independent randomly chosen cell sections, meaning each analysed section stems from a different cell.
We have added the following sentence to the methods to clarify this issue: "Sections from the central part of the cell (large diameter) and that contained organelles were imaged from only the central section in a serial section ribbon, to ensure that no cells were imaged in duplicate.". The number of 37 cell volumes was calculated by multiplying the section thickness (70nm) by the number of sections imaged overall (2143 sections) and dividing it by the average thickness of a cell (4µm), to give an idea of how large volume of cell that have been analysed. However, we realise that the addition of this information causes more confusion than clarification, so we have removed it from the manuscript. With this information, we hope the reviewer agrees with us that the analysis is fine unaltered.

2. "It is unclear how cell and organelle size were determined from EM cross-sections. For sphere-like 3-D objects, the cross-sectional area will be highly dependent on whether the section is taken closer to the object's equator or to its poles. Is there a method for choosing which cross-section is representative of the overall object? If so, this should be added to the text. If not, this would reflect a major weakness with the image analysis. In a related question, the authors present analyses in Fig 7 comparing organelle to cell size. This seems to assume that the same cross-section provides an accurate relative measurement for both the organelle and cell, but this would be highly dependent on how the cell and organelle are arranged. Again, the authors should clarify how cell and organelle size ratios were derived."

We have clarified the process of how sections were chosen in the Materials & Methods section (see above). Although we cannot be entirely certain how close in to the equator of the cell we are, we hope that with the amendment above to the Methods, it becomes clear that we are looking at approximately the central third of the cell. Additionally, we are using large numbers of cells (300+ per timepoint) for better statistics. This is mostly a problem with larger organelles, such as the vacuole, which is also why we have confirmed the observed trends with light microscopy.

3. "Given that many organelles consist of multiple individuals (i.e. vacuole and mitochondria), reporting only on the average individual size of these organelles seems insufficient, and that a total size of all of the same type of organelle should also be analyzed. Unfortunately, if a single cell has multiple copies of the same organelle, it's unclear how to create a combined size from measurements of cross-sectional area."

Thank you for pointing out more constructive ways to use our data! We have added a figure (figure S6), where the sum of the organelle size in absolute numbers, as well as the sum of the organelle size divided by the number and area of cells are displayed respectively.

4. "The interpretation of the change in electron density in the vacuole as relating to acidification was interesting, but seemed under-supported. The authors provide a correlative argument that electron density matches the intensity of the BCECF dye in fluorescence experiments, and from this assert a connection. But the mechanism of this seems highly speculative. Is there other evidence or a reference that uranyl acetate staining depends on pH? It seems possible that the change in electron density would signify a different in the protein/nucleic acid/lipid concentration in the vacuole, have the author's ruled out this possibility? Further testing of this possibility would be helpful."

© 2021. Published by The Company of Biologists under the terms of the Creative Commons Attribution License (https://creativecommons.org/licenses/by/4.0/).

To test our hypothesis further, we tried to find a yeast mutant with a more acidic pH than wt, which we would then predict to have a higher percentage of electron dense vacuoles. In a publication they screened for such mutants (Brett *et al*, 2011), so we selected a row of their most acidic mutants and stained them with Quinacrine. Unfortunately, we could not replicate their findings that these mutants have a more acidic vacuole than wt and could therefore not test this as specifically as we intended.

Instead, we have added an experiment where electron micrographs of cells lacking the protease prb1 were quantified. We added this to the discussion:
"Since we show here that UA staining has a potential to be used as a pH indicator in cells, it is important to rule out the possibility that the UA staining of vacuoles was only influenced by their biomolecular content. UA binds to phosphate and carboxyl groups of molecules (Hayat, 2000). Therefore, it normally stains proteins and nucleic acids in cells (Reynolds, 1963; Hawes *et al*., 2007). Toward this end, we used two different mutants, *prb1Δ,* which has more acidic vacuoles and *vma2Δ,* which has deacidified vacuoles compared to wt. The acidic vacuoles of *prb1Δ* were more electron dense (Figs 3E, S1B), while the more basic vacuoles of the *vma2Δ* mutant were electron translucent (Fig. 3E). Both of these mutants are inhibited in their proteolytic activity (Zubenko and Jones, 1981), which should yield a higher biomolecular content of their vacuoles. If the presence of biomolecules such as proteins and nucleic acids alone influenced the vacuolar staining, these mutants would not have exhibited such different staining patterns, which supports our hypothesis that UA preferentially stains an acidic environment."
We also weakened our statement to that our method could *potentially* be used to visualize pH changes.

5. "The authors conclude that nucleus-vacuole contacts are increased, based on the relatively large increase in N-V contact length beyond what is expected compared to the size increases observed for the vacuole. However, it's not readily apparent what the expected change should be here. Do the authors have a geometric model to explain their expectation? Is the expectation based on the percent increase in the vacuole cross-sectional area? How is the change in cell size incorporated into this estimate? This would seem to provide a major constraint for organelle organization."

Thank you for this comment, we have verified our findings in accordance with the model that we have developed and used to determine the expected length of a contact site, and adapted the manuscript accordingly. We have added markings in the supplementary figure S5A and B for the expected change in contact size in relation to the sizes of the respective organelles. The model on how those expected values are calculated is explained in the Materials & Methods section:

"The contact site fraction of an MCS, *CSF*, represents the fraction of total adjacent cell circumferences that is an MCS and was calculated as follows for time points *t*:

$$CSF(t) = \frac{MCS_{abs}(t)}{circum_{organelle1}(t)+circum_{organelle2}(t)}.$$

At the beginning of the heat shock time course (*t=0*), the ratio of the respective median values is *CSF0* and can be used as indicator for the expected length of an MCS, using the respective median values: $MCS_{expect}(t) = \mathrm{CSF}_0 \times \left(circum_{organelle1\,(t)} + circum_{organelle2\,(t)}\right)$."

The total cell size is not included in this model, however we have added supplementary information showing the relationship between the total organelle and total cell area (Figs S5C, D). These two graphs both show the proportion of the cell area occupied by a certain organelle and the change of this proportion throughout the heat shock time course.

6. "The authors show the rise of electron dense structures in the nucleus/mitochondria, and electro translucent structures in the cytoplasm. Further discussion on these might be useful, in particular, are the EDC's in the nucleus related to the nucleolus?"

Thank you for your interest in these structures. We have now pointed out nuclear structures seen in EM, based on previous literature (Hyde, 1965; Karreman *et al*, 2009; Thelen *et al*, 2021) and described how these differ from EDC. EDCs have previously been assumed to be protein aggregates in HS cells (Panagaki *et al*, BioRxiv, 2021). Only 10% of the EDCs were found in the nucleolus, not indicating a preferred localization of protein aggregates to this structure. We have added a note about this in the text (row 194) and also added a graph as supplementary figure S2.

© 2021. Published by The Company of Biologists under the terms of the Creative Commons Attribution License (https://creativecommons.org/licenses/by/4.0/).

7. "It's unclear why in different statistical analyses, it seems like multiple timepoints were grouped together (examples, Fig 2B, 2C, 4B, 4E, 6C.) Furthermore, the groupings are inconsistent, and the data for some timepoints seems to be used in multiple statistical comparisons, which seems problematic. Please provide an explanation for how data for different timepoints were determined to be grouped together for statistical analysis."

Thank you for pointing out this ambiguity in the graphs. Different timepoints were not grouped together for statistical analysis. This confusion may stem from the presentation of significances within graphs, which we hope has now been made clearer both within the figures themselves and the figure legends. Further, p-value tables have been added to the supplementary materials.

Reviewer 2

Reviewer 2 Advance Summary and Potential Significance to Field:

"This is the first systematik ultrastructural analysis of cells in response to mild heat shock. This data can/will serve as a basis for further functional and structural studies."

Reviewer 2 Comments for the Author:

"In this paper, the authors have analyzed ultrastructural changes in budding yeast in response to mild heat shock. This is a nice systematic study on this topic, and I have only some minor comments:"

1. "The authors mention the importance of the heat shock phenomenon in neurodegenerative human diseases. Why is it then useful to study the cytological changes by using yeast as a model? What is the motivation here to use budding yeast? This aspect should be introduced to the interested reader."

Thank you for this encouragement to ponder on the larger picture of our work, we have amended the introduction to include this and further references.

"Heat shock in budding yeast (*Saccharomyces cerevisiae*) is extensively used as a model to study neurodegenerative human diseases (Winderickx *et al.*, 2008; Kaliszewska *et al.*, 2015), where inclusion bodies of aggregated misfolded proteins accumulate at specific sites in the cytoplasm and nucleus (Takalo *et al.*, 2013; Chung, Lee and Lee, 2018). Examples of such diseases are Alzheimer's, Huntington's, and Parkinson's (Tenreiro *et al.*, 2013). The processes involved in the genesis of these diseases are heavily influenced by proteins' temporal and spatial interactions, both of which can be suitably studied using budding yeast (DebBurman *et al.*, 1997; Schirmer and Lindquist, 1997)."

2. "This comment is also related to the section Materials & Methods. Cells were "randomly" chosen. I think the procedure should be explained in more details. Did the authors, for instance, cut serial sections? If so, how did the authors exclude multiple countings of the same cell?

The reviewer is correct that this is unclear as reviewer #1 had a similar concern. The clarification is given above in response to Reviewer #1, but we repeat the information here for your convenience:
"Thank you for pointing out this ambiguity in the manuscript. Indeed, the 2143 micrographs correspond to 2143 independent randomly chosen cell sections, meaning each analysed section stems from a different cell.
We have added the following sentence to the methods to clarify this issue: "Sections from the central part of the cell (large diameter) and that contained organelles were imaged from only the central section in a serial section ribbon, to ensure that no cells were imaged in duplicate." The number of 37 cell volumes was calculated by multiplying the section thickness (70nm) by the number of sections imaged overall (2143 sections) and dividing it by the average thickness of a cell (4µm), to give an idea of the relation between section thickness and the size of a yeast cell.

© 2021. Published by The Company of Biologists under the terms of the Creative Commons Attribution License (https://creativecommons.org/licenses/by/4.0/).

However, we realise that the addition of this information causes more confusion than clarification, so we have removed it from the manuscript. With this information, we hope the reviewer agrees with us that the analysis is fine unaltered.”

3. "In Figure 1B, the lower mid image is rather dark, and it is hard to see the cytological details. Another image should be selected."

We have selected a different image with a clearer view of cytological details.

4. "In general, it should be avoided to put letters directly on the marked organelles (for instance the ‘m’ for the mitochondria directly on the organelle). This way, information is hidden for the reader."

We have checked the placement of each label and moved them to ensure as little as possible cytological information is hidden from the reader.

5. "As for figure 2B, I am not sure about the presentation of the data. The authors should think about presenting the average of all three experiments. The distribution could be given in a suppl. figure."

We have chosen to present all three experiments separately to highlight the scope of the study. Additionally, it allows to see patterns within the data (such as the values of the 5 min timepoint of experiment three being consistently smaller across cell and organelle size), without obscuring any information. For clarity, we have added the median of each timepoint in the graphs as grey bar.

6. "I’m also not sure about the grouping of times 0-15 and 30-90 min for the statistical analysis and testing. What is the rationale behind this grouping?"

Different timepoints were not grouped together for statistical analysis. This confusion may stem from the presentation of significances within graphs, which has now been made clearer both within the figures themselves and the figure legends. Further, p-value tables have been added to the supplementary materials.

7. "As for figure 3A, has the density of the vacuoles been evaluated/grouped by ‘eye’?"

Indeed, the vacuole densities were evaluated by eye by the same person, in relation to the electron density of the cytoplasm.

8. "For Fig 4D, could the authors show more examples of the mitochondria in a suppl. figure?"

Yes, we have added further examples of mitochondria in the supplementary figure S2.

9. "Personally, I think Figure 7A-B should be shifted to the results section. I think this part of the figure is misplaced. Fig. 7C could stay in the discussion, as a kind of summary."

Figure 7A-B and have been re-arranged the figures and the text accordingly.

10. "The matching up in pairs is a very interesting result. The authors should comment on this. What could be the biological message here? I would like to hear the opinion of the authors on this."

It seems that the pairs of organelles that match up in their change in size have complementary or co-dependent functions in the heat shock response. We have now discussed our ideas further in the manuscript.

11. "The first sentence of the discussion: change ‘allows’ to ‘allowed’."

Thank you! Corrected.

© 2021. Published by The Company of Biologists under the terms of the Creative Commons Attribution License (https://creativecommons.org/licenses/by/4.0/).

12. "I think the part about the sample preparation for electron microscopy should be expanded. For the protocol on yeast filtration, the original paper for this procedure should be cited. As for the use of the resin, what are the details for infiltration with HM20 and the polymerization of the resin? How was this done? Why did the authors use this specific resin for their experiments? I guess, the clear visibility of the membranes in resin was the argument here."

Our lab uses HM20 resin exclusively as it gives great preservation of cell morphology in combination with our freeze substitution protocol and offers the possibility of performing immuno-gold labelling on the same sample whenever necessary. We have elaborated on the sample preparation in the Materials & Methods section and added relevant citations.

13. "As for the selection of the cells for quantification, see my comment above."
[Replied to above in comment 2.]

We thank the reviewers for their time and effort to improve this manuscript!

Second decision letter

MS ID#: JOCES/2020/258325

MS TITLE: Large Organellar Changes occur during Mild Heat Shock in Yeast

AUTHORS: Katharina S Keuenhof, Lisa Larsson Berglund, Kara L Schneider, Sandra Malmgren Hill, Per O Widlund, Thomas Nyström, and Johanna L Höög

ARTICLE TYPE: Research Article

I am happy to tell you that your manuscript has been accepted for publication in Journal of Cell Science, pending standard ethics checks. Thank you for submitting this interesting manuscript to JCS.

Reviewer 1

Advance summary and potential significance to field

The manuscript's findings were summarized in the original review and are unchanged in this revision.

Comments for the author

The authors have made key clarifications and analysis to improve their manuscript and is now suitable for publication.

Reviewer 2

Advance summary and potential significance to field

Comments for the author

I now agree with all the provided corrections. From my side, this paper is now ready for final acceptance.

© 2021. Published by The Company of Biologists under the terms of the Creative Commons Attribution License (https://creativecommons.org/licenses/by/4.0/).
